# Carbon dioxide tolerability and toxicity in rat and man: A translational study

**DOI:** 10.3389/ftox.2022.1001709

**Published:** 2022-10-13

**Authors:** Rutger van der Schrier, Monique van Velzen, Margot Roozekrans, Elise Sarton, Erik Olofsen, Marieke Niesters, Chantal Smulders, Albert Dahan

**Affiliations:** ^1^ Department of Anesthesiology, Leiden University Medical Center, Leiden, Netherlands; ^2^ Department of Anesthesiology, Noordwest Ziekenhuisgroep, Alkmaar, Netherlands; ^3^ Shell Global Solutions International BV, Hague, Netherlands

**Keywords:** carbon dioxide, CO_2_ transport, CO_2_ storage, tolerability, toxicity, translational study

## Abstract

**Background:** Due the increasing need for storage of carbon dioxide (CO_2_) more individuals are prone to be exposed to high concentrations of CO_2_ accidentally released into atmosphere, with deleterious consequences.

**Methods:** We tested the effect of increasing CO_2_ concentrations in humans (6–12%) and rats (10–50%) at varying inhalation times (10–60 min). In humans, a continuous positive airway pressure helmet was used to deliver the gas mixture to the participants. Unrestrained rats were exposed to CO_2_ in a transparent chamber. In both species regular arterial blood gas samples were obtained. After the studies, the lungs of the animals were examined for macroscopic and microscopic abnormalities.

**Results:** In humans, CO_2_ concentrations of 9% inhaled for >10 min, and higher concentrations inhaled for <10 min were poorly or not tolerated due to exhaustion, anxiety, dissociation or acidosis (pH < 7.2), despite intact oxygenation. In rats, concentrations of 30% and higher were associated with CO_2_ narcosis, epilepsy, poor oxygenation and, at 50% CO_2_, spontaneous death. Lung hemorrhage and edema were observed in the rats at inhaled concentrations of 30% and higher.

**Conclusion:** This study provides essential insight into the occurrence of physiological changes in humans and fatalities in rats after acute exposure to high levels of CO_2_. Humans tolerate 9% CO_2_ and retain their ability to function coherently for up to 10 min. These data support reconsideration of the current CO_2_ levels (<7.5%) that pose a risk to exposed individuals (<7.5%) as determined by governmental agencies to ≤9%.

## 1 Introduction

Carbon dioxide (CO_2_) is a product of the aerobic metabolism of energy containing nutrients (carbohydrates) in humans and animals and from industry- and transport-related combustion processes. The rise in global CO_2_ emission (37 gigatons in 2018)^1^ and consequently its accumulation in the atmosphere caused and continues to cause a global rise in temperature, which has deleterious effects on the climate (Jackson et al., 2018; Loeb et al., 2021). Hence, there is the need for CO_2_ reduction, for example through capture and storage (Marchetti, 1977; O’Callaghan, 2018). CO_2_ is captured in industrial plants and transported *via* pipelines to underground (or undersea) storage facilities, such as depleted gas stores (O’Callaghan, 2018). Large scale implementation of this technique in the near future could result in storage of CO_2_ in the vicinity of populated areas. In case of incidents (*e.g.*, pipeline failures and/or problems at storage facilities) point source releases of large quantities of CO_2_ will result in a cloud with high CO_2_ levels. Acute exposure to high levels of CO_2_ may be hazardous for human health, both within the fence line (workers), as well as outside the fence line (general public). CO_2_ exerts its acute toxicity through different mechanisms. Most importantly, at acute exposure to high levels (>30%), CO_2_ induces the displacement of oxygen (CO_2_ is heavier than air), causing a hypoxic environment and toxicity from asphyxia (the lack of oxygen combined with an increase in arterial CO_2_ concentrations). Second, upon inhalation, CO_2_ causes sympathoexcitation and acidosis (Lumb, 2017), which may cause arrhythmias and tissue injury. Finally, CO_2_ induces severe anxiety and fear due to cerebral acidosis (Griez et al., 2007; Ziemann et al., 2009), which may cause inability to take coherent decisions, an effect that is further aggravated by cognitive decline at high inspired CO_2_ concentrations (Sayers et al., 1987).

Since its discovery in 1754, several scientific publications were dedicated to the effects of CO_2_, but the number of dose escalation studies on the effect of acute exposure to CO_2_ on changes in human physiology is sparse (Loevenhart et al., 1929; Sayers et al., 1987; Griez et al., 2007; Gill et al., 2014). The highest inhaled CO_2_ concentration studied was 40% and published almost a century ago (Loevenhart et al., 1929). While these extremely high concentrations were only applied for a few breaths, it did provide some information on CO_2_-induced subjective symptoms. Here we present data from an exploratory and translational project, performed in humans and rats, that was aimed to improve our understanding of the physiological and behavioral effects of short-term (acute) exposure to high levels of carbon dioxide. This understanding will facilitate the safety design and emergency response procedures for CO_2_ transport and storage facilities. We exposed healthy young adult humans to 6–12% inhaled CO_2_ for 10–60 min, and exposed rats to 10–50% CO_2_ for up to 60 min.

Since there is a lack of systematic examination of the effect of time-varying escalating concentrations of inhaled CO_2_ in humans and translation between man and animal (rodent) studies is sparsely described, we performed the current study, with ultimate aim to provide data to reassess the current guidelines for CO_2_ exposure. To these ends, we determined the effect of CO_2_ inhalation on tolerability (in human and rats), lethality (in rats) and on the acute acid-base state as measured by arterial pH. Studies involved regular arterial blood sampling for blood gas analyses during and following exposure to CO_2_ and objectivation of behavioral changes. In humans, we further tested cognition, cerebral oxygen saturation, and measured hemodynamic parameters. In the animals, the lungs were examined to assess pathology and possible causes for CO_2_-related death. Next, to correlate the results of the animal with those of the human studies, we developed a translational model of pH to provide insight in the effect of higher CO_2_ concentrations than tested in our human study population on pH. We hypothesized that at least 50% of healthy volunteers are able to tolerate 9% CO_2_ inhalation for periods up to 30 min.

## 2 Materials and methods

### 2.1 Ethics and registration

In this exploratory project both animal and human studies were performed. The animal protocol was approved by the University Animal Ethics Committee (Leiden University, Leiden, the Netherlands), the human protocol by the Human Ethics Committee (Commissie Medische Ethiek, Leiden, the Netherlands) and the Central Committee on Research Involving Human Subjects (CCMO, competent authority) in The Hague, the Netherlands, all in 2015. During the study we remained in close contact with the ethics committee and regularly reported on the progress and occurrence of adverse events of the study. The human protocol was registered in the trial register of the Dutch Cochrane Center (www.trialregister.nl) under identifier NL4955 on 1 August 2015. Since the trial register is no longer available, the protocol can be obtained from the authors (a.dahan@lumc.nl). The study was conducted in accordance with current Good Clinical Practice Guidelines and adhered to the principles of the Declaration of Helsinki. The study was performed from 1 Oct 2015-1 March 2018.

### 2.2 Study in humans

#### 2.2.1 Participants

Healthy male volunteers were recruited to participate in the study. Inclusion criteria were age 18–25 years; body mass index in the range 18–25 kg/m^2^ (inclusive) with body weight between 50 and 100 kg; absence of any significant medical, neurological, or psychiatric illness as determined by the investigators; and willingness and competence to sign a written informed consent. Exclusion criteria were: a history of panic disorder; a history of hypertension; present or a history of any illicit drug use; present or a history of alcohol abuse (intake of more than 4 units per day); smoking of more than 10 cigarettes per day; participation in a drug trial in the 3 months prior to screening; any physical abnormality as determined by an independent physician; or any other issue/condition that in the opinion of the investigator would complicate or compromise the study or the well-being of the subject (these include claustrophobia, fear of needles, car sickness, recurrent headaches, tinnitus, unwillingness to follow the instructions of the researchers).

#### 2.2.2 CO_2_ exposure and stopping rules

All subjects were studied once (*i.e.* they only were exposed to one concentration of CO_2_ for just one duration). The first set of 54 subjects received one of three inhaled carbon dioxide concentrations, 6%, 7.5% or 9%, with adjusted inspired oxygen levels of 19.7%, 19.3% and 18.9%, respectively (these values simulate O_2_ displacement by CO_2_). Exposure times were escalating with 6 subjects inhaling 6% CO_2_ for 10 min, 6 others for 30 min and finally 6 others for 60 min. When no stopping rules were met, we increased the CO_2_ concentration to 7.5% and again exposed 6 subjects for 10 min, 6 others for 30 min and finally 6 subjects for 60 min. After successful completion of these cohorts, we amended the protocol to further expose 20 volunteers to 10% CO_2_ (inspired O_2_ 18.8%; *n* = 10) and 12% CO_2_ (inspired O_2_ 18.4%; *n* = 2) for a maximum duration of 10 min. Stopping rules were: pH < 7.20, heart rate >180 beats per min, systolic blood pressure >200 mmHg, diastolic blood pressure >120 mmHg or subjectively experienced side effects warranting discontinuation (as decided by the subject or the investigators). In case stopping rules were met in cohorts 1 to 10, we discontinued the experiment in that subject but continued to the next subject; however, the study would be terminated when all subjects of a specific cohort would have reached stopping rules. For cohort 11, we decided to evaluate the advancement to a next subject based on the results of the previous subject.

#### 2.2.3 Monitoring

To ensure the safety of the subjects and obtain as much information during CO_2_ exposure we continuously measured the ECG, peripheral arterial oxygen saturation through a finger probe (SpO_2_), and regional cerebral oxygen saturation (rSO_2_) using an INVOS^TM^ 5100C cerebral oxygen saturation monitor (Medtronic, Minneapolis, MI, United States) with the sensor applied to the left side of the forehead. An intravenous access line was placed in the cubital vein of the non-dominant arm, and a 22G arterial cannula was placed in the radial artery (at the level of the wrist) of the non-dominant arm. The arterial line was connected to a FloTrac sensor and Vigileo system (Edwards Lifesciences, Irvine, United States) for cardiac index monitoring. The oxygen and carbon dioxide concentrations were measured in the helmet close to the mouth using a capnograph (Capnomac Ultima, Datex-Ohmeda, Finland).

#### 2.2.4 Gas delivery

An adult size continuous positive airway pressure helmet (Dimair®) was used to deliver the gas mixture to the participants. The helmet was positioned over the head and closed around the neck, and allowed normal verbal communication with the research staff and permitted performance of cognition tasks without any constraints or respiratory efforts. A gas mixture was delivered to the helmet from a custom-build computer-controlled gas-mixing setup (the first-generation Leiden gas mixer) containing three mass flow controllers (Bronkhorst High-Tech BV, Veenendaal, the Netherlands) for delivery of 50 L/min gas in any combination of O_2_, CO_2_ and nitrogen (Dahan et al., 1990). The helmet had an outlet (Ø 44 mm) ensuring adequate drainage of gas flow and guaranteeing no pressure buildup within the helmet even at high respiratory rates. Prior to the exposure to increase levels of CO_2_, the subjects breathed a gas mixture that mimicked room air (20.8% O_2_ in nitrogen).

#### 2.2.5 Study sequence

After a period of relaxed breathing, the following baseline values were collected: arterial gas values (pH, arterial PO_2_, arterial PCO_2_ and oxygen saturation; derived from the i-STAT blood gas analyzer (Abbott, United States) using CG8+ and CG4+ cartridges; in the 10 and 12% exposure cohorts, two devices were used to keep up with the frequent blood sampling; the device is able to measure pH values from 7.7—6.5), cardiac index, cerebral oxygen saturation (rSO_2_), and subjective experiences (sedation, nausea, headache) and a p-deletion test. Next, the subject was exposed to the preset gas mixture. During exposure arterial blood gas measurements were obtained at 5-min intervals (2-min intervals for the 10 and 12% CO_2_ cohorts), subjectively experienced side effects at 10-min intervals and non-invasive blood pressure using an arm cuff at 10-min intervals. All other variables (O_2_ and CO_2_ concentrations in the helmet, cardiac index and brain oxygen saturation) were logged continuously at 50 Hz.

The CO_2_ exposure ended when the intended duration of the experiment was reached, in case stopping rules were met, upon request of the subject, or upon judgement of the attending physician. Upon termination, 100% oxygen was administered for 5 min; thereafter the helmet was removed and the subject breathed room air. In case the experiment ended prematurely, an attempt was made to obtain a final arterial blood gas sample was obtained. We queried the subjects immediately after CO_2_-exposure for side effects including sedation, nausea and headache using a 11-point Likert scale ranging from 0 (not present) to 10 (maximum experience of symptom); querying continued at 30-min intervals for at least another hour. After removal of the helmet, the subject was monitored for 60-min. The subject was dismissed from the laboratory only if side effects (including subjective effects) had waned and the attending physician agreed that the subject was sufficiently recuperated to go home.

#### 2.2.6 The p-deletion test

To determine the cognition of the subject during and following CO_2_-exposure, the modified p-deletion test was performed (Dixon and Thornton, 1973). The test consists of 19-lines on one page of 38 lower case letters b, d, q and p. A total of 45 letters p are distributed at 2 to 3 per line at a random location within the line. The test was performed at t = 10-, 30- and 60-min during CO_2_ exposure (depending on the cohort) and at t = 30- and 60-min following CO_2_ exposure. The test was scored by determining the number of successfully completed lines and the number of mistakes.

### 2.3 Study in rats

#### 2.3.1 Animals and study design

CD® (Sprague Dawley) IGS adult male rats (250–270 g) were purchased from Charles River Laboratories (Leiden, the Netherlands). The animals were obtained with a femoral arterial catheter in place (Instech Laboratories Inc., PA, United States). The animals were exposed to one of five inspired CO_2_ concentrations, 10, 20, 30, 40 or 50%, with adjusted inspired oxygen concentrations of 18.9, 16.7, 14.6, 12.5 or 10.4%, respectively, in cohorts of 8 animals (total number of animals used in the study is 41, including 1 control animal breathing just ambient air). Each animal was exposed for 60 min, after which they were euthanized by pentobarbital injection.

The arterial line allowed continuous access to arterial blood (100 μl) for blood gas analysis and glucose and [K^+^] measurement. In order to restrict blood loss from the animals two distinct sampling strategies were applied per cohort. The sampling regimen of the first group (*n* = 4) focused on acute changes in blood gas values following the initiation of CO_2_ exposure (t = 0) with sampling at 2-min interval from t = 0 (just prior to exposure) to t = 10 min, and 2 final samples at t = 15 and 20 min. In the second group, the remaining 4 animals, sampling occurred at t = 0, 5, 10, 20, 30, 40, 50 and 60 min following the start of CO_2_ exposure. Maintenance of the arterial catheter prior to the study was according to the guidelines of the Charles River laboratories. During exposure, all animals were monitored for changes in behavior and respiratory rate was counted at 5-min intervals. Animals that appeared in discomfort (*e.g.* because of epileptiform activity) or moribund (*e.g.* gasping) prior to the end of the CO_2_ exposure were taken out of the inhalation box and euthanized.

CO_2_ exposure occurred in a custom-build Perspex transparent inhalation box. The animals were unrestrained throughout the experiment. The air humidity and temperature in the box were maintained constant by using of a humidifier. The box was connected to the first-generation Leiden gas mixer, and the desired gas mixture flowed through the box at 20 L/min. Each CO_2_ exposure was preceded by inhalation of a gas mixture that mimicked ambient air. The arterial line was accessible from the outside of the box. Gas concentrations within the box were constant until completion of the 1-h CO_2_ exposure or in case the animal became moribund and was removed from the box. Following the death of the animals, macro and micro-pathological examination of the lungs was performed to determine exposure-induced lung damage by the department of animal pathology of the Leiden University Medical Center.

#### 2.3.2 Macroscopic and microscopic inspection of the lungs

Obduction and macroscopic analysis was performed in all animals. A microscopic inspection of the lungs was performed in a random selection of the animals (*n* = 7), exposed to 10% CO_2_ (*n* = 1), 20% CO_2_ (*n* = 1), 30% (*n* = 1), 40% CO_2_ (*n* = 1) and 50% CO_2_ (*n* = 3). To serve as control, one additional animal was euthanized with pentobarbital after 1 h of air breathing (without CO_2_ exposure). First, the lungs were inspected for subpleural hemorrhages. Next, five to six lung sections were stained with hematoxylin and eosin to grade the number of hemorrhages (alveolar, peribronchial, perivascular), alveolar or perivascular emphysema and % total emphysematous area. A total of 46 observations were included in the analysis.

### 2.4 Data analysis

For sample size calculation, we focused on the tolerability of inhaling 6% CO_2_ versus 9% CO_2_ in the human population and assumed that all subjects in the 6% CO_2_ arm of the study would tolerate 30 min of CO_2_ inhalation, while in the 9% CO_2_ arm this would be 5. We then calculated a minimum sample size of 8 to detect whether the stated difference exists between the two proportions. Given the uncertainties in the assumptions, we included 10 subjects in each group (Wang and Chow, 2007). The data are described as mean ± SD, median (range) or number (percentages). No formal data comparisons were performed as this was an exploratory study. Additionally, group numbers were small, comparisons were hampered by data loss from discontinuations (human study) or premature death (animal study) and concentration-effect relationships were evident, making a *post hoc* comparison less relevant.

### 2.5 Translation between species

The translational model starts out with the CO_2_ alveolar mass balance (Olofsen et al., 2010):

$${VOL}_{ALV}\times \frac{{dPCO}_{2}}{dt}=\dot{V}\times \left(P_{I}{CO}_{2}-P_{ALV}{CO}_{2}\right)+k\times Q\times \left(C_{v}{CO}_{2}-C_{ALV}{CO}_{2}\right)$$ (1)

where VOL_ALV_ = is the volume of alveolar tissue, $\dot{V}$ is minute ventilation, P_I_CO_2_ is the inspired CO_2_ concentration, P_ALV_CO_2_ is the alveolar CO_2_ concentration (we assume that this is equal to arterial and end-tidal CO_2_ concentration), k is a constant that relates blood CO_2_ content to concentration, Q cardiac output (minus pulmonary shunt), C_V_CO_2_ the CO_2_ content of venous blood, and C_ALV_CO_2_ the alveolar CO_2_ content, which we assume equals arterial CO_2_ content. To simplify the calculations, we further assume that VOL_ALV_ is negligible over the time scale of interest, ventilation and cardiac output rapidly reached their increased values and remained constant, CO_2_ concentration is linearly related to CO_2_ content in blood, venous CO_2_ concentration has a first-order delay relative to arterial CO_2_ concentration, the production of CO_2_ is negligible relative to the high inspired CO_2_ concentration, and [HCO_3_^-^] in blood is constant. Then:

$$\dot{V}\times \left(P_{I}{CO}_{2}-P_{ALV}{CO}_{2}\right)+k\times \left(P_{v}{CO}_{2}-P_{ALV}{CO}_{2}\right)=0$$ (2)

where P_V_CO_2_ is the venous CO_2_ concentration. Mixing of CO_2_ in the lungs was described as follows:

$$P_{ALV}{CO}_{2}=\alpha \times P_{I}{CO}_{2}+\beta \times P_{v}{CO}_{2}$$ (3)

where α and β are mixing parameters with α = $\dot{V}$ /( $\dot{V}+k)$ and β = 1—α. Since CO_2_ production is not included in the model, the sum of α and β might differ from 1.

The CO_2_ mass balance of the body is modeled as:

$$\frac{dP_{v}{CO}_{2}}{dt}=\left(P_{ALV}{CO}_{2}-P_{v}{CO}_{2}\right)\times \phi$$ (4)

where *ϕ* is the body CO_2_ equilibration rate constant.

The Henderson-Hasselbalch equation equals:

$$pH=6.1+{[}^{10}\log{HCO}_{3}^{-}]/(0.23\times P_{ALV}{CO}_{2}))$$ (5)

CO_2_ exposure was tested as a covariate in the log domain of all parameters. All pH-time data of the human and rat data were fitted simultaneously to the model, while simulations were performed to predict the pH-time data in humans inspiring 10, 15, 20, 30, 40 and 50% CO_2_. The analysis and simulations were performed in NONMEM, a software package for nonlinear mixed effects modeling, using a population approach.

## 3 Results

### 3.1 Study in humans

**FIGURE 1:**
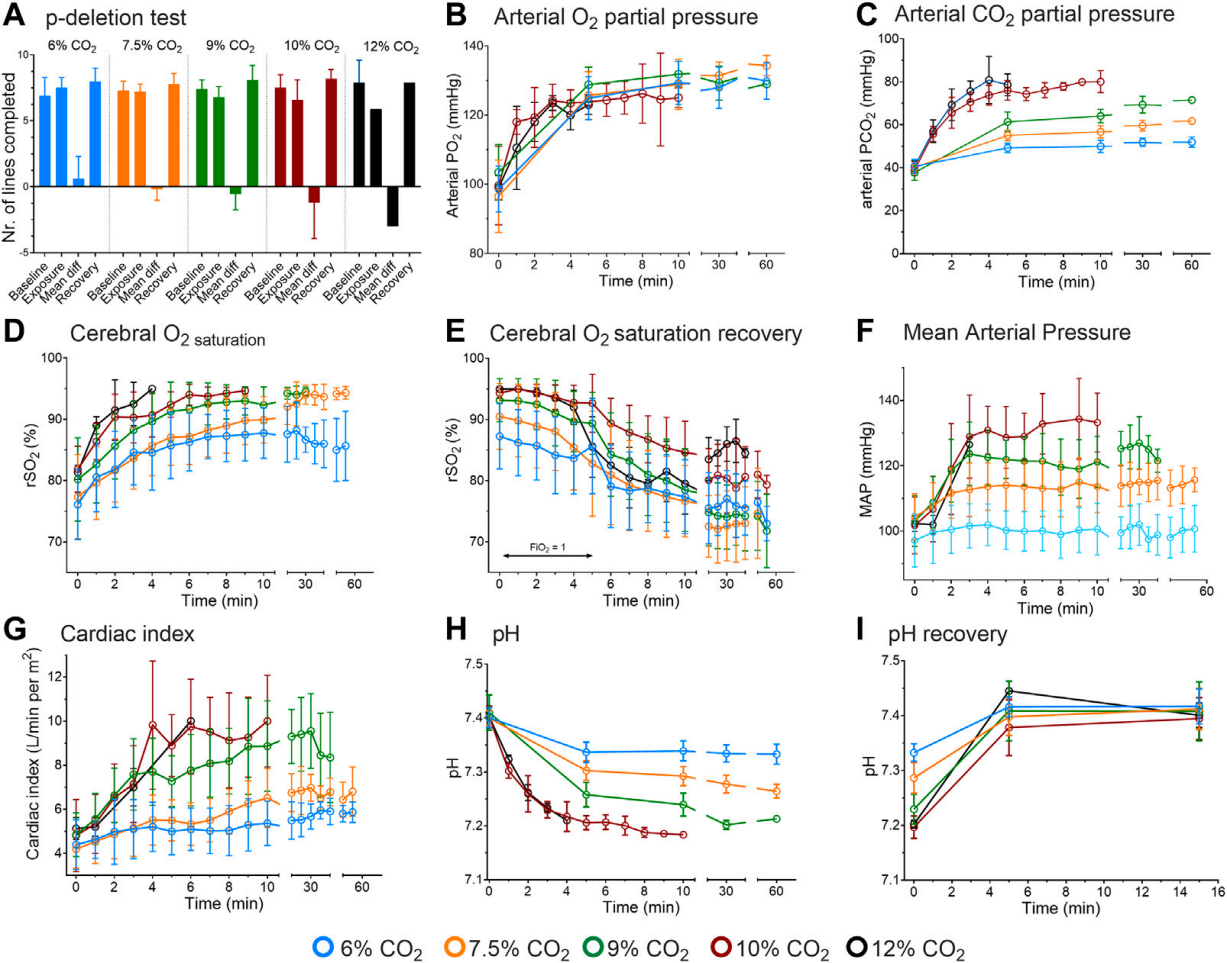
Results of the human experiments. **(A)** Cognitive function assessed by p-deletion test. Results are from before CO_2_ inhalation, during inhalation and during recovery. The mean difference is the difference between tests obtained at baseline and during inhalation. **(B)** Arterial oxygen concentrations during 6–12% CO_2_ inhalation. **(C)** Arterial carbon dioxide concentrations during CO_2_ inhalation. **(D)** Cerebral oxygen concentration as measured by near-infrared spectroscopy during CO_2_ inhalation. **(E)** Cerebral oxygen concentration following CO_2_ inhalation while breathing 100% oxygen (for 5 min) and next while breathing room air. **(F)** Mean arterial pressure during CO_2_ inhalation. **(G)** Cardiac index during CO_2_ inhalation. **(H)**. pH values during 6–12% CO_2_ inhalation. **(I)**. pH data obtained during the first 15-min following CO_2_ exposure while breathing 100% oxygen. The data obtained at 12% inhalation are from 2 subjects; duration of 10 and 12% CO_2_ inhalation was max. 10 min. All data are mean ± SD.

We intended to include seventy-four male subjects in the study. The study had eleven specific CO_2_ inhalation cohorts. Cohorts 1–9: 6, 7.5 and 9% CO_2_ inhalation for 10, 30 and 60 min (with 6 subjects in each cohort) and after finalizing cohorts 1-9, cohorts 10 and 11 (included after ethics approval of a protocol amendment): 10 and 12% inhalation for 10 min, with 10 subjects per cohort. The actual number of treated subjects was 66, as the study was prematurely terminated after 2 subjects were exposed to 12% CO_2_ for less than 10 min because of side effects (see below). The mean (±SD) age and mean body mass index of the treated subjects were 24 ± 3 years and 23 ± 2 kg/m^2^, respectively. Among the cohorts no differences were observed in age or body mass index distributions. After the start of CO_2_ exposure, the intended inspired values were reached within 1 min, arterial PCO_2_ and PO_2_ are given in Figures 1B,C.

#### 3.1.1 CO_2_ tolerability

Average inhalation times are given in Table 1. The inhalation of 6 and 7.5% CO_2_ was well tolerated for up to 60 min by all subjects. The next cohort, 9% CO_2_, was well tolerated by subjects inhaling the gas mixture for 10 min. Longer exposure was less well tolerated by 4 (out of 6) subjects that completed 30 min and 1 of 6 completed 60 min of inhalation. The causes of discontinuation were anxiety, panic or exhaustion due to heavy breathing. In the last 2 cohorts (#10 and 11), 3 of 10 subjects completed the 10-min inhalation of 10% CO_2_, while none of the subjects completed the 10-min inhalation of 12% CO_2_ (only two subjects were tested in this cohort). The causes of discontinuation were dissociation, blackout, anxiety and overwhelming dyspnea. The two subjects that were discontinued in the 12% CO_2_ inhalation (after 7 ± 2 min) had pH values <7.2 and were unable to communicate with the investigators although they appeared awake. Additional symptoms observed in the 9, 10 and 12% cohorts were myoclonic twitches, restlessness, headache (average score ±SD 4.8 ± 2.2 on an 11-point Likert scale) and sedation (2.5 ± 1.9 on an 11-point Likert scale); these symptoms were not the reason for participant-initiated discontinuation. After consultation with the ethics committee, we decided not to proceed to a third subject in the 12% CO_2_ cohort and prematurely ended the study. Interestingly, upon recovery, during inhalation of 100% oxygen, some subjects developed a headache, and some of these subjects vomited. We relate this to the occurrence of sudden vasoconstriction (due to hyperoxia) and cerebral hypoperfusion following maximal vasodilation (due to hypercapnia) (Kety and Schmidt, 1948).

**TABLE 1:** Inhalation of carbon dioxide in humans.

	
Inhaled concentration	6%			7.5%			9%			10%	12%
Intended duration (min)	10	30	60	10	30	60	10	30	60	10	10
Number of subjects that completed inhalation/total number of participants	6/6	6/6	6/6	6/6	6/6	6/6	6/6	4/6	1/6	3/10	0/2*
Actual inhalation duration (min)	10 ± 0	30 ± 0	60 ± 0	10 ± 0	30 ± 0	60 ± 0	10 ± 0	16 ± 12	20 ± 22	7 ± 3	7 ± 2

*Just 2 of the 10 planned subjects entered this cohort, after which the study was prematurely terminated. Inhalation duration: mean ± SD.

#### 3.1.2 CO_2_ effect on cognition and brain oxygen saturation

Cognitive performance, as measured by the p-deletion test, was dose-dependently affected by CO_2_ inhalation, with the worst performance at inhaled CO_2_ concentrations of 10 and 12%, with respect to number of lines completed (Figure 1A) and number of errors (data not shown). These effects proved to be transient as a swift recovery was observed upon termination of the exposure. Inability to focus and the laborious breathing activity were the main causes for the decrease in performance. The decreased cognitive performance was unrelated to the oxygen concentration in the brain as brain oxygen concentration (measured by near-infrared spectroscopy on the forehead) increased from 75 to 80–90% within 10 min of inhalation (Figure 1D; recovery of rSO_2_ given in Figure 1E), most probably related to an increase in cerebral blood flow associated with the increase in cardiac output and cerebral vasodilation.

#### 3.1.3 CO_2_ effect on hemodynamics variables

From 6 to 10% CO_2_ exposure, mean arterial pressure and cardiac index dose-dependently increased to a maximum of 140 mmHg and 10 L/min per m^2^, respectively (Figures 1F,G). No further increase in mean arterial pressure was seen in the 12% CO_2_ cohort, which we relate to the small number of subjects (*n* = 2) that remained in that cohort and the frequent blood sampling that limited the ability of the invasive blood pressure measurement device to obtain reliable blood pressure values.

#### 3.1.4 CO_2_ effect on blood gas values and estimated minute ventilation

Arterial PO_2_ increased over the first 5 min of exposure to 125 mmHg, in a dose-independent manner (Figure 1B). This was unexpected as at constant inspired oxygen fraction, water pressure and atmospheric pressure, alveolar PO_2_ is inversely related to alveolar PCO_2_. At higher inspired CO_2_ levels alveolar PO_2_, and subsequently arterial PO_2_ and oxygen saturation should decrease. Additionally, the right-shift of the hemoglobin-oxygen dissociation curve supports oxygen unloading from hemoglobin, causing a further drop in arterial PO_2_. Possibly a reduction of the ventilation/perfusion mismatch, related to an increase in ventilation and cardiac output, counteracted the expected decrease in arterial PO_2_ (see also below).

In all cohorts, pH decreased rapidly to a plateau after 5 min of CO_2_ inhalation (Figure 1H). Dose-dependency was apparent until the 10% CO_2_ cohort with no further decrease in the 12% CO_2_ cohort, which we again attribute to the small number of subjects that remained in that cohort. Lowest pH was 7.18 in the subject that was subsequently discontinued from the 12% CO_2_ cohort. Upon recovery, pH returned to baseline values within 5 min; pH recovery is given in Figure 1I. Finally, glucose levels (only measured in the 6–9% CO_2_ cohorts) remained constant over time within the normal range (range 5.5–6.3 mmol/L with normal values 4.0–6.1 mmol/L).

From the measured arterial PCO_2_ values, we were able to estimate the minute ventilations at static pH values as observed in the 6–9% CO_2_ cohorts, using the formula of the hypercapnic ventilatory response, V_E_ = S × (PaCO_2_—B), where V_E_ is minute ventilation, S the slope of the hypercapnic ventilatory response, PaCO_2_ arterial PCO_2_ and B the extrapolated arterial PCO_2_ at zero ventilation (Nielsen and Smith, 1951). We used values of S and B estimated in similar study populations (Dahan et al., 1990; Dahan et al., 2007; Algera et al., 2022). The results were for 6% CO_2_ an estimated minute ventilation of 29 L/min, for 7.5% CO_2_ 48 L/min and for 9% CO_2_ 66 L/min.

### 3.2 Study in rats

Forty adult male Sprague Dawley rats with mean weight 261 ± 33 g were exposed to increasing concentrations of inhaled CO_2_ (10, 20, 30, 40 and 50%) with 8 animals per dosing cohort. Additionally, 1 animal served as control for pathology.

#### 3.2.1 CO_2_ tolerability

All sixteen animals completed the 1-h exposure to 10 and 20% CO_2_ without serious discomfort. The animals in the 10% CO_2_ cohort exhibited normal behavior throughout the 1-h exposure. The animals in the 20% CO_2_ cohort displayed hyperactivity and excitation for the first 30–40 min of exposure and then transitioned slowly into hypoactivity during the remaining time in the inhalation box. Hyperactivity consisted of rapid irregular breathing, disorganized behavior and uncoordinated movements. During exposure to 30% CO_2_, an initial 10-min period of hyperactivity was followed by pronounced hypoactivity (apparent CO_2_ narcosis); in four animals sudden and severe epileptiform activity occurred and the animals were, as we wanted to prevent any further discomfort, immediately euthanized with pentobarbital. None of the other four animals showed any excitatory signs or irregular breathing. During exposure to 40% CO_2_, an initial 1–2 min period of hyperactivity was followed by complete hypoactivity and rapid shallow regular breathing (apparent CO_2_ narcosis); none of the animals died or displayed signs of epilepsy. During exposure to 50% CO_2_, apparent narcosis rapidly developed and the animals showed slow shallow breathing. Five animals died after 14–25 min of exposure, the others survived until the end of exposure.

#### 3.2.2 CO_2_ effect on respiratory rate and blood gas values

**FIGURE 2:**
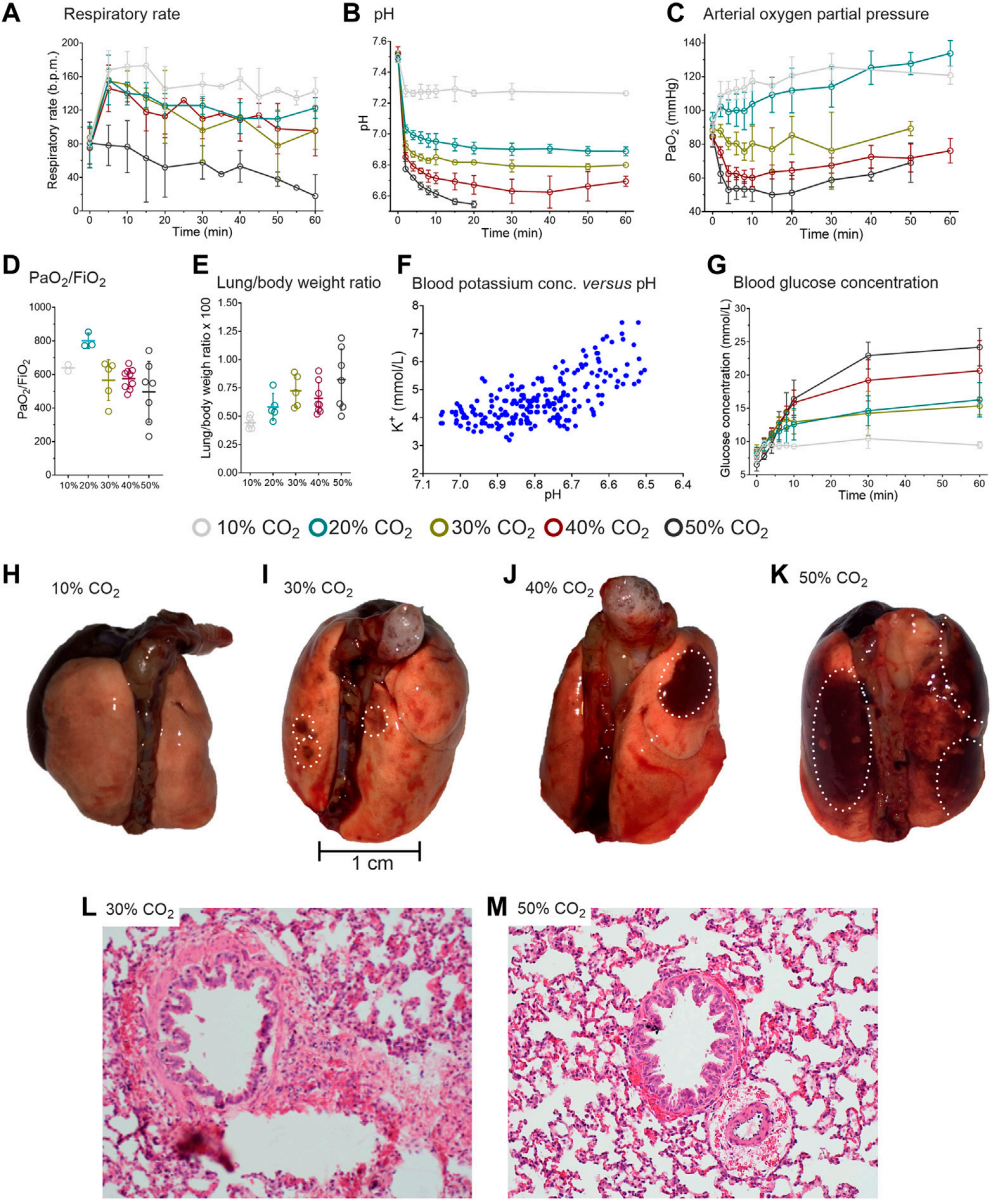
Results of the rat experiments. **(A)** Respiratory rate during the 1-h inhalation of 10, 20, 30, 40 or 50% CO_2_. **(B)** pH during CO_2_ inhalation. During 50% CO_2_ inhalation 5/8 animals died after 14–25 min. In the remainder of animals, no pH samples were obtained beyond 20 min **(C)** Arterial oxygen concentrations. **(D)** Arterial PO_2_/FiO_2_ ratio in the different CO_2_ exposure groups. **(E)** Lung/body weight ratio in the different CO_2_ exposure groups. different CO_2_ exposure groups. **(F)** Blood potassium concentration versus pH. **(G)** Blood glucose concentration in the various CO_2_ exposure groups. **(H-K)**. Macroscopic aspect of the lungs of three distinct animals that inhaled 1-h of 10%, 30% and 50% CO_2_. The white circles indicate the location of hemorrhages. l and m. Microscopic aspect of lung sections of two distinct animals treated with 30% **(I)** and 50% **(M)** CO_2_. Peribronchiolar and perivascular hemorrhages are present as well as emphysema throughout the section. The slices in panels l and m are 200-times magnified and stained with hematoxylin and eosin. The data in panels a-c and g are mean ± SD, in panels d and e median (50–75% interquartile) are given.

During 10–40% CO_2_ inhalation, respiratory rate increased from baseline values (80 breaths/min) to 150–175 breaths/min within 10-min of CO_2_ exposure (Figure 2A). At 50% CO_2_ inhalation, a decrease in respiratory rate was observed from baseline to 25 breaths/min. In all cohorts, the decline in pH was biphasic, with an initial rapid decrease followed by either no further decline (10% CO_2_ cohort) or a further slower decline (Figure 2B). The magnitude of acidosis development was dose-dependent with lowest pH values observed in the 10% CO_2_: pH = 7.26 ± 0.2 at t = 10 min, 20% CO_2_ cohort: pH = 6.90 ± 0.4 at t = 30 min, 30% CO_2_ cohort = 6.82 ± 0.01 at t = 20 min; 40% CO_2_ cohort: pH = 6.63 ± 0.08 at 30 min; and in the 50% inhalation cohort: pH = 6.54 ± 0.02 at t = 20 min. Due to missing data from the loss of animals, device-related limitation to measure pH below 6.5 or issues with sampling from the arterial line, these values may be an overestimation of the actual values in the highest CO_2_ cohort.

In the low CO_2_ inhalation cohorts (10 and 20%) arterial oxygen concentrations increased, despite reduced FiO_2_ values that were implemented to simulate O_2_ displacement by CO_2_ (Figure 2C) (Gill et al., 2002). Similar to the observations in humans, we expect that this is the consequence of the reduced ventilation/perfusion mismatch (*i.e.* reduced shunting), the increase in breathing frequency, hypercapnia-potentiated hypoxic pulmonary vasoconstriction and increased cardiac output. The increase in arterial PO_2_ was maintained throughout the 60-min of CO_2_ inhalation.

In the 30–50% CO_2_ inhalation cohorts, arterial oxygen concentrations significantly decreased (Figure 2B). Despite the pulmonary damage (see below), the PaO_2_/FiO_2_ ratio remained unaffected by the CO_2_ level with relatively normal to supra-normal values irrespective of the inhaled CO_2_ concentration (Figure 2C). Due to missing data from the loss of animals in the 30 and 50% CO_2_ inhalation cohorts and sometimes sampling issues, the PaO_2_/FiO_2_ ratios are most probably largely overestimated.

#### 3.2.3 CO_2_ effect on potassium and glucose concentrations

Extracellular potassium concentration increased with decreasing pH due to the H^+^/K^+^ exchange across the cell membrane (Figure 2F). The increase in plasma K^+^-concentration may be associated with cardiac arrhythmias and death in the animals that succumbed to high dose CO_2_ inhalation, although other causes of death are not excluded (see below). A dose-dependent increase in glucose concentration was observed with the highest glucose concentration measured in the 50% CO_2_ cohort (24 mmol/L; Figure 2G). No increase in glucose was observed in the 10% CO_2_ cohort.

#### 3.2.4 CO_2_ effect on macroscopic and microscopic changes to the lungs

Macroscopic inspection of the lungs revealed that the animals exposed to 30–50% CO_2_ had signs of subpleural or pulmonary hemorrhage and edema (Figures 2H–K). The lung to body weight ratio dose-dependently increased indicative of accumulation of fluid and blood in the lungs (Figure 2E). An important finding was that the animals that had prematurely expired had 60% greater lung weights than animals that completed the exposure: 2.4 ± 0.3 g versus 1.4 ± 0.2 g. Microscopically, a dose-dependent increase in emphysematous pulmonary changes, edema and hemorrhages was observed (Table 2). The microscopic changes observed in the 30% and higher CO_2_ cohorts were severe and most probably not compatible with (long-term) survival (Figures. 2L and 2M). These findings indicate that pulmonary damage may be the final and definite cause of death from CO_2_ and/or acute hypoxia in these rats (at 50% CO_2_ the arterial PO_2_ is around 75 mmHg, Figure 2C).

**TABLE 2:** Effect of CO_2_ on lung damage in rats.

CO_2_^***^ (sample size)	Hemorrhages*				Emphysema**	Perivascular edema and emphysema*
	Alveolar	Peri-bronchial	Peri-vascular	Sub-pleural		
Control (1)	0 (0–0)	0 (0–0)	0 (0–0)	0 (0–0)	30%	1 (1–1)
10% (1)	0 (0–1)	0 (0–0)	1 (1–1)	0 (0–0)	30%	0 (0–0)
20% (1)	0 (0–1)	0 (0–0)	0 (0–0)	0 (0–0)	30%	1 (1–2)
30% (1)	0 (0–0)	0 (0–0)	1 (1–1)	0 (0–1)	55%	2 (2–2)
40% (1)	0 (0–0)	0 (0–0)	1 (1–1)	0 (0–0)	65%	2 (2–2)
50% (3)	2 (1–2)	3 (1–3)	3 (2–3)	2 (1–2)	83%	3 (3–4)

*Median number of observations per lung section (range); ** % total emphysematous area.

***The data are obtained from 5-6 lung sections with 1 animal per inhalational level, except for 50% CO_2_ inhalation, where the results of 3 animals are given.

### 3.3 Translation between species

**FIGURE 3:**
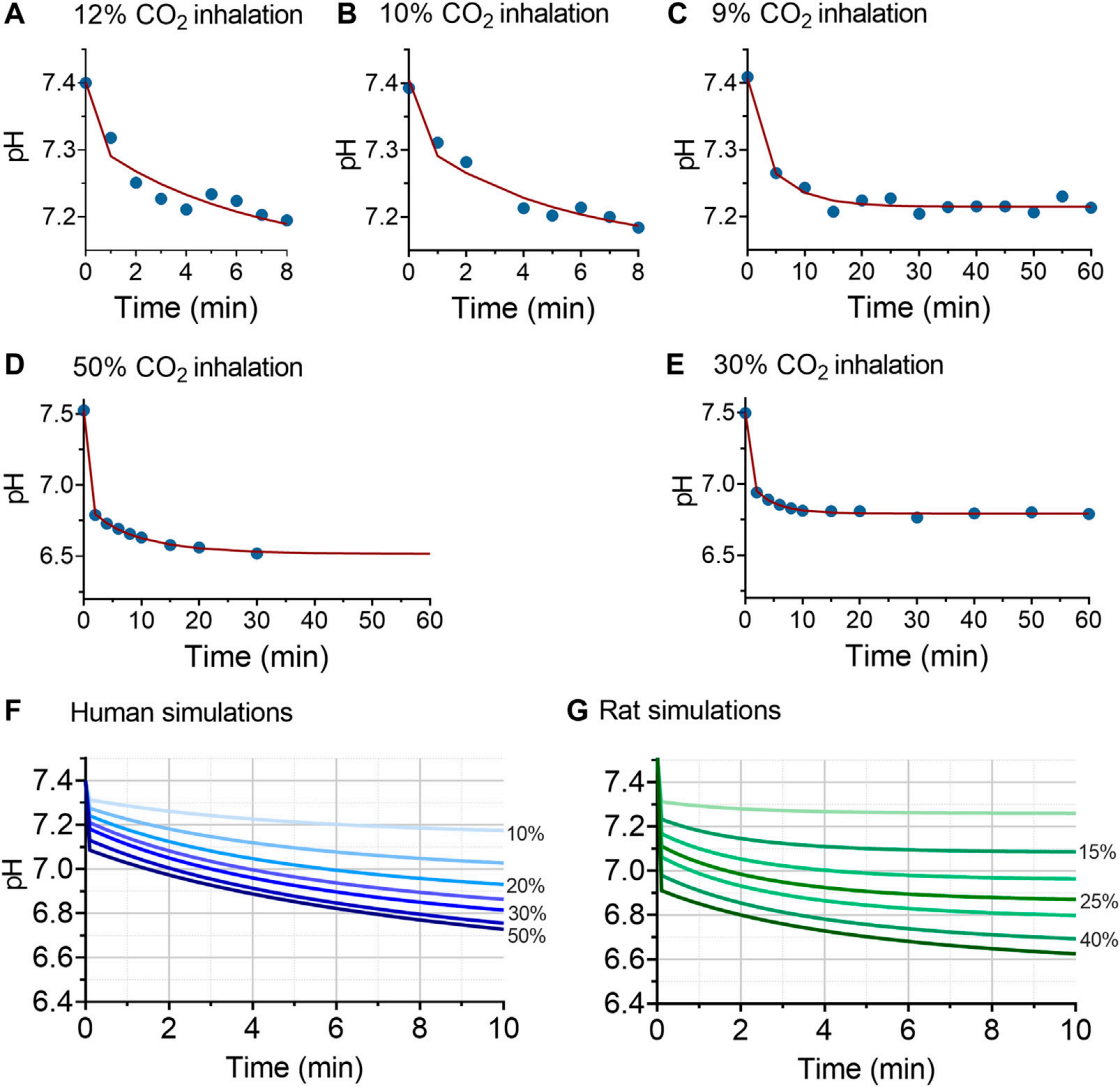
Results of the translational model analysis of acidosis (pH). Panels **(A–E)** given examples of the pH model fits in humans **(A–C)** and **(D and E)** in rats. The blue circles are the measured data, the red lines the data fits. Panels **(F and G)** give the simulations at inhalation values of 10% (top lines in the two panels), 15%, 20%, 25%, 30%, 40% and 50% (bottom lines in the two panels) CO_2_ inhalation. The human simulations indicate that at inhalation levels of 15% CO_2_ or higher, pH values <7.2 are readily reached.

In order to predict the pH effect of higher CO_2_ inhalations than tested in the human subjects, we constructed a translational physiological model of acidity (pH). To that end, we simultaneously analyzed the human and animal data, using a model that combined the CO_2_ mass balance in the alveoli, the CO_2_ mass balance in the body compartment and the Henderson-Hasselbalch equation using a population approach in the statistical package NONMEM. Inspection of the data fits as well as of the goodness of fit plots (data not shown) indicate that the data from two species were well described by the model. Examples of data fits are given in Figures 3A–E: Figures 3A–C give examples human data fits with inhalations of 12, 10 and 9% CO_2_; Figures 3D,E show two examples of rat data fits. Parameters estimates were similar for rat and human data, except for CO_2_ mixing parameters α and β. Parameter values (median ±standard error of the estimate) at baseline, prior to any CO_2_ exposure, were: [HCO_3_^-^] = 25.6 ± 0.21 mmol/L, PCO_2_ = 47.3 ± 0.83 mmHg and the body CO_2_ equilibration rate constant *ϕ* = 1.98 ± 0.56; for humans α = 0.89 ± 0.01 and β = 1—α, for rats α = 0.67 ± 0.12 and α + β = 0.93 ± 0.007. CO_2_ exposure was a significant covariate on *ϕ* with logϕ = -0.031 ± 0.007. These results indicate a slower mixing of CO_2_ in humans with a slower decrease in pH over time and reduction of *ϕ* at higher CO_2_ exposures.

Predictions of pH values at 10% inspired CO_2_ or higher are given in Figures 4F (humans) and 4g (rats). The human simulations predict that pH will decrease below 7.2 after 6 min of 10% CO_2_ inhalation and within 2 min after 15% CO_2_ inhalation. At higher inhaled CO_2_ concentration, pH decreased below 7.2 within 1 min of exposure. For example, at 20% inhaled CO_2_ pH decreased to 7.25 within 50 s, and reached a value of 6.9 after 10 min of inhalation. At 50% CO_2_ inhalation pH decreased initially to 7.08 and further towards 6.72 after 10 min of inhalation. The decrease in pH was slower in humans than in animals at similar simulated CO_2_ inhalations_,_ which we related to slower CO_2_ mixing in humans than rats, as reflected by differences in values for α and β between the two species.

## 4 Discussion

The main findings of this translational project were that in a population of heathy young volunteers, the inhalation of 6 and 7.5% CO_2_ was well tolerated for up to 1-h, while tolerance to 9% CO_2_ was limited to the short exposure time (10 min). Longer duration of 9% CO_2_ inhalation and higher CO_2_ concentrations (10 and 12%) were not tolerated, with causes for discontinuation: exhaustion, anxiety, acidosis (pH < 7.2, which was one of the stopping rules), dissociation or blackout (both led to the inability to communicate with the subject). The oxygenation of the subjects remained intact with an increase in arterial PO_2_ and brain oxygen saturation. In rats, all animals survived the 10% and 20% CO_2_ inhalation, while at 30% CO_2_, 4 animals developed epileptiform activity and 5 animals died during 50% CO2 exposure. These deaths were related to the high CO_2_ aggravated by the presence of hypoxia, and were associated with severe lung damage, sympathoexcitation (as deduced from the blood glucose levels) and possibly also acidosis-induced hyperkalemia. Oxygenation of the animals worsened at higher CO_2_ concentrations with a reduction in arterial PO_2_ to about 50 mmHg at 50% CO_2_ inhalation. We were able to connect the human and rat data by constructing a translational model of pH allowing the prediction of pH values over time over the CO_2_ concentration range of 10–50%.

The difference in the CO_2_-level at which the human volunteers and the rats lost tolerance to further exposure was evident but CO_2_-related physiological changes were such that we could perform an interpolation between species. Irrespective of species, we may conclude that up to 20% CO_2_ inhalation, arterial PO_2_ increases and only decreases at higher inhalation levels. Possibly the initial increase in arterial PO_2_ is related to an increase in cardiac output, reduced pulmonary vascular resistance, CO_2_-induced bronchodilation, acidosis-related improvement of hypoxic pulmonary constriction, that all combined led to an improvement of the ventilation/perfusion ratio. These effects counteract the expected reduction in arterial PO_2_ because of the lower inspired oxygen faction (reflecting oxygen dispersion by CO_2_), right-ward shift of the oxygen dissociation curve, increased dead space ventilation due to tachypnea, and recruitment of poorly perfused lung areas. At higher inhaled CO_2_ concentrations, the decrease in arterial PO_2_ is explained by these later factors and by lung damage (hemorrhages, edema and emphysema, Table 2 and Figure 2I–2M). The maintained PaO_2_/FiO_2_ ratio, particularly in the higher CO_2_ inhalation cohorts, was not expected, but we relate these to that fact that measurements were restricted to animals that survived with possibly less pulmonary damage (attrition bias). Additionally, the FiO_2_ values in our experiments were low, in contrast to the high FiO_2_ in ventilated patients with respiratory distress disorders. Another relevant observation was the development of hyperglycemia that occurred at CO_2_ concentrations of 10% and higher. Hyperglycemia is a sign of severe stress and is also observed during hemorrhagic shock and critical illness, and is related to sympathoadrenergic activity (Marik and Bellomo, 2013; Scheen et al., 2021). Importantly, hyperglycemia is related to poor outcome in critical illness (Scheen et al., 2021). The absence of hyperglycemia in the human experiments may be related to the restriction of measurements to the 3 lowest CO_2_ concentration cohorts. Overall, these results indicate the validity of translational studies in the extrapolation of physiological responses from one species (rat) to the other (human), in this case the response to high inhaled concentrations of CO_2_.

The development of a translational model to describe pH values at higher inhaled CO_2_ than tested in our human population was successful. The results (Figure 1H, Figure 2B, Figure 3F,G) indicate that the decrease in pH was more rapid in rats than in humans upon initiation of inhalation, a steady-state in pH developed earlier in the rat, and buffering capacity at steady state was greater in the rat. We relate this in part to a faster CO_2_ mixing (as reflected by the species differences in model parameters α and β, see Eq. 3), possibly related to the smaller body size and relatively higher spontaneous ventilation and cardiac output in the rats. The greater tolerability to CO_2_ in the rat may be an indication of the large phylogenetic distance between rat and man, in contrast to the distance between rat and other animals with a high tolerability to hypercapnia such as the naked mole rat (Husson and St. John Smith, 2018; Park et al., 2021; Pamenter and Cheng, 2022). In our human study, we did not allow continuation of CO_2_ inhalation when pH values decreased below 7.2. This was an arbitrary level, as we are aware that lower pH levels (pH < 6.9) are sometimes observed in humans without deleterious consequences (Goldstein et al., 1990). Still eventually extreme pH values (pH < 6.6) do compromise cellular and protein function and consequently will affect cardiac and brain function (Poole-Wilson, 1982; Rehncrona, 1985; Orchard and Kentish, 1990).

The causes for discontinuation (apart from the low pH observed in two subjects during 12% CO_2_ inhalation and death in the rats inhaling 50% CO_2_) were related to neuropsychiatric effects with dissociation, blackout, anxiety or exhaustion in the human subjects, and epileptiform activity during CO_2_ narcosis in the animals. Several mechanisms may be involved in the inability to tolerate CO_2_. Cerebral hyperperfusion, increased intracranial pressure, cerebral edema and/or encephalopathy may have occurred during CO_2_ inhalation. To detect possible structural brain damage in the animals, we removed the brains of the animals at obduction for a gross examination but observed no signs of edema or hemorrhage (data not shown). Possibly this is related to the fact that arterial PO_2_ remained above 75 mmHg throughout the hypercapnic exposure (Paljärvi et al., 1982; Yang et al., 2016). Still, we cannot exclude some deleterious effect of CO_2_ on the function of specific brain centers at levels up to 12% CO_2_ in humans and at higher concentrations in the animals. The coupling between local blood flow and synaptic activity may be severed at extreme levels of hypercapnia possibly due to a CO_2_ effect at the acid sensing ion channels (ASICs), which are important in regulating the coupling between local blood flow and synaptic activity (Faraci et al., 2019; Stark et al., 2019). Further studies are needed to disentangle the complex interaction of arterial and brain tissue CO_2_ concentrations and reversible and irreversible cerebral damage.

The injury to the rat lungs was of such severity at the 30% and higher CO_2_ cohorts that they were considered (eventually) lethal. Still, we need to realize that a small part of the emphysema may have been related to the pentobarbital injection as also in the control animal some emphysema was observed (Table 2). Irrespective, these results suggest that the pulmonary damage was the main cause of death of the animals in the 50% CO_2_ inhalation cohort. This is also consistent with and aggravated by the effects of acute hypoxia as oxygen levels at 50% CO_2_ were around 10.5% (approx. 75 mmHg; Figure 2C) (Appelt et al., 2021). However, we cannot exclude other contributing factors such as heart failure, cardiac arrhythmias or cerebral damage. Our findings agree with earlier animal studies in which acute exposure to 40% CO_2_ in oxygen in 8 rats was associated with dyspnea and fulminant pulmonary edema in all animals (Brosnan et al., 2007).

The combined rat and human data enable human risk assessment for CO_2_ transport and storage facilities, where CO_2_ is stored or transported in large quantities. In addition, the data can support emergency response measures for these facilities. When individuals are exposed to an excess of CO_2_ in ambient air, an important question is at what levels of CO_2_ inhalation does the human body maintain its ability to adequately function to, for example, escape from the incident scene or to perform a cognitively challenging task. An answer to this question is not only dependent on the results of our current study in healthy young volunteers but is certainly also dependent on the age and more importantly the physical condition of the exposed individual as well as presence of underlying cardiac or pulmonary disease. Our current results indicate that during exposure to 9% CO_2_ the body retains its ability to function for 10 min, albeit with large variability in tolerance with some subjects able to withstand 30 min and one subject 60 min of exposure. Still at this inhaled concentration all subjects experienced some form of discomfort, anxiety and reduced cognitive performance. Hence, it remains questionable whether at this inhaled concentration, the individual will be able to coherently perform a complex task. We expect that fleeing the scene will remain possible. Note that we expect CO_2_ tolerance to decrease rapidly in older individuals with lower resilience and those with existing cardiac or pulmonary disease. The translational model predicts that at inhaled CO_2_ concentrations greater than 9%, pH will rapidly decrease to values that further hamper the capacity to function adequately.

Risk assessment for CO_2_ storage and transport facilities also includes estimates of probability of incidents, and probability of death in these incidents. Probability of death is typically estimated from acute lethality data in animals or anecdotal information form incidents. To support risk assessment for CO_2_ storage and transport facilities, CO_2_ lethality in rats has been investigated by Muijser et al. (2014). Their lethality data are consistent with the data presented in this paper. When considering only rat lethality as endpoint, the CO_2_ dose-response curve is too steep to allow for derivation of a reliable estimate of probability of death in humans. Our study has included additional physiological parameters to allow translation of the animal data to the human situation. Human tolerability was demonstrated here to levels of 9% CO_2_ for short durations (up to 10 min) and these data support a reconsideration of the current CO_2_ risks determined by *e.g.* the United Kingdom Health and Safety Executive and United States Environmental Protection Agency, which mention the unconsciousness can result within a few minutes of exposure to 7% CO_2_ (US Environmental Protection Agency, 2000; McGillavray and Wilday, 2009). Further analysis of the data in this study could provide essential insight into the probability of human effects and fatality after acute exposure to high levels of CO_2_ and will drive land-use planning, setting of risks management measures, and emergency response planning.

Finally, in laboratory animals, a CO_2_ overdose is the most commonly used practice for euthanasia (Conlee et al., 2005; Boivin et al., 2017; Turner et al., 2020). Our rat data indicate that this will coincide with various neurological symptoms, indications of stress and tissue damage. Additionally, CO_2_ at high concentrations activates nociceptors in the nasal cavity which is associated with severe pain sensations (Thürauf et al., 1991; Green and Hummel, 2013; Hickman et al., 2016). Hence, from an animal welfare perspective it is questionable whether this form of euthanasia is harmless as our findings as well as those of others indicate that a CO_2_ overdose is highly distressful and will cause damage to lung tissue (Conlee et al., 2005; Boivin et al., 2017; Turner et al., 2020).

## Data Availability

The data are available from the authors after agreement has been obtained regarding purpose of analysis and protocol.
